# A review of product safety regulations in the European Union

**DOI:** 10.1365/s43439-022-00057-8

**Published:** 2022-06-17

**Authors:** Jukka Ruohonen

**Affiliations:** University of Turku, Turku, Finland

**Keywords:** Safety, Consumer protection, Harmonization, Standards, Liability, Literature review, European Union, GPSD

## Abstract

Product safety has been a concern in Europe ever since the early 1960s. Despite the long and relatively stable historical lineage of product safety regulations, new technologies, changes in the world economy, and other major transformations have in recent years again brought product safety to the forefront of policy debates. As reforms are also underway, there is a motivation to review the complex safety policy framework in the European Union (EU). Thus, building on deliberative policy analysis and an interpretative literature review, this paper reviews the safety policy for nonfood consumer products in the EU. The review covers the historical background and the main laws, administration and enforcement, standardization and harmonization, laws enacted for specific products, notifications delivered by national safety authorities, recalls of dangerous products, and the liability of these. Based on the review and analysis of these themes and the associated literature, some current policy challenges are further discussed.

## Introduction

Safety has reached a global priority in the face of the global COVID-19 crisis. Yet safety—understood in the present context as a risk to human health—has long been on the agenda of legislators around the world, including those in the European Union. Although global pandemics—from the Spanish flu through the swine flu to the present crisis—have often captured the attention in popular discourse, law-backed preparations for hazardous accidents have been implemented from the 1960s onward. These preparations also address concerns that range from terrorism, crime, and radicalization to traffic, pollution, and environmental hazards, all of which may also include safety consequences [6, 40]. Interestingly, many of the incidents that originally prompted the preparations have been forgotten or buried in history books. The production, storage, and transport of chemicals is a good example: the global and European safety legislations for chemicals moved forward through crises; dioxin in 1976, toxic oil syndrome in 1981, methyl isocyante in 1984, Amerithrax in 2001, and so on and so forth [17]. Consumer products do not cause such large-scale accidents, but the safety consequences from these often affect more humans than individual hazardous accidents. The regulation of safety requirements for consumer products is also particularly difficult.

Product safety is a subset in the larger jurisprudence of consumer protection. Throughout the world, the rationale builds upon the economic and information asymmetries between producers and consumers; the latter are in a weaker position with respect to the former, both with respect to bargaining power and knowledge [74]. As the history of the automobile industry vividly demonstrates, the incentives of producers to blindly pursue profits have resulted in many dangerously defective or even hazardous products that have put consumers’ lives at risk [68]. Given that later on safety became an important competitive benefit in the car industry [61], black and white perceptions should be avoided, as always. Nevertheless, the general rationale of product safety regulations is to protect the weaker party. A particular focus has historically been placed upon vulnerable groups, such as children, the disabled, and the elderly [52]. This protection rationale appeared on the legislative agenda already in the early 1960s both in Europe and in the United States [38]. It did not take long for it to also appear in the European Economic Community; the early safety directives were passed in the early 1970s.

However, fragmentation has prevailed to the present day. Although perhaps not as visibly as in some other areas of consumer protection, European product safety legislations have also suffered from fragmentation and incoherence. A partial explanation originates from the domain; it is difficult to legislate consumer goods and services due to the pace of innovation and technological progress. But another partial explanation stems from different cultures and historical trajectories; the power struggles between the organized interests of consumers and producers affected regulatory traditions differently in different member states [1]. A similar struggle between organized interests has affected consumer law at the EU level [56]. A further partial explanation can be found among producers; there have often been diverging or even strictly competing interests between sectors and producers, and their locations in different member states and regions [16]. Likewise, there have been occasional arguments that the EU’s safety regulations are used for protectionist objectives [38], particularly against Chinese products [35]. Concerns such as environmental consequences have intensified the struggles.

These challenges translate into research challenges; the EU’s product safety policy has always been notoriously complex and difficult to understand. This complexity provides a motivation for the present short review on the key legislations in Europe. But there is a further motivation: reforms are already underway for European product safety legislations due to the rapid technological progress and its impact upon the economy and consumers with it. Electronic commerce, data, platforms, and other ingredients of contemporary economy have also changed the incentives, externalities, and asymmetries between producers and consumers [34, 37]. Although product safety does not perhaps weigh as much as security and privacy in these new circumstances, it is not difficult to imagine also new safety risks arising from, say, artificial intelligence, robotics, and cyber-physical systems, along with many other technological development trends. Indeed, many novel but wretched safety incidents have already occurred and been cataloged for artificial intelligence applications [48]. Of these incidents, the legacy of Elaine Herzberg is perhaps the most memorable and the saddest example; she was the first pedestrian killed by a self-driving car.

## Concepts, Approach, and Data

A few preliminary remarks are in order before the actual review. A few basic concepts should be clarified, some words should be said about the approach to the review, and something should be noted about the empirical data used alongside the review for a few illustrative points.

### Concepts

Safety, security, hazard, and risk—among many related concepts—are domain specific, debated, and generally ambiguous terms [6]. In terms of information security, which is a subset of a larger security conundrum, a risk is sometimes understood as a probability that an attack occurs; other times it is more specifically seen as a conditional probability resulting from a threat and a vulnerability. From this perspective, protection of (information) security implies protection against intentional attacks, whereas safety is more about unintentional lapses [40]. Given the context of consumer products, it is also useful to frame unintentional harm to those that have consequences for the health and well-being of humans. This framing aligns with the concept of hazard, which is often understood merely as a potential source of harm [61]. Despite the differences, for illustrative purposes, the basic information security concepts can be translated to the safety context: a vulnerability could be a defect in a product that exposes a safety threat to human health, such as, say, a suffocation, a strangulation, or a serious electromagnetic disturbance. In this review, as well as in the EU regulations, such safety threats of consumer products exclude social, psychological, and related factors with potential health consequences. It is also important to emphasize that the particular regulations considered exclude food products, medicine and drugs, and occupational health risks, among other things.

Product safety is presumed to be the earliest case of a risk-based approach to regulation in the EU. Since the 1990s, safety regulations have relied on a precautionary principle: dangers to health and environment should be taken into account through systematic, scientifically based risk analysis [67]. Although definitions vary across domains, a risk-based approach according to the European safety regulations is seen to generally cover three dimensions: risk assessment, risk management, and risk communication [59]. These vary from one product to another. For many products—from chemicals to cosmetics, risk assessments may involve rigorous laboratory testing. For some other products, including software products, assessments range from the following of standards, documentation, and sound engineering practices to quality controls and safety verification [9, 51, 57]. Risk management, likewise, varies across products. For many products, including both tear-and-wear hardware products and software products, life-cycle management is usually present; a product should be safe throughout its intended life in the hands of consumers [61]. It should also be stressed that risk management is not only strictly about safety; for producers, cost–benefit analysis is often present as well [38, 67]. In the European Union, risk communication carries a particular weight: whenever a risk is found from a product, producers should communicate the risk to public authorities and consumers. With these clarifications of the basic terminology in hand, the approach taken for the review can be briefly elaborated.

### Approach

The review approach taken follows the tradition of practice-oriented policy analysis. Unfortunately—just like with safety, there are no commonly agreed on definitions for policy analysis. Roughly, policy analysis revolves around the questions of what, how, and why governments do what they do, and what difference it makes [18]. In this short review, the focus is on what they do with a policy. This policy refers to a set of European legislations and standards designed to ensure the safety of nonfood consumer products. Safety in itself is seen as the primary (but not necessarily the only) answer to the why question.

Then, it seems reasonable to maintain that most practitioners of policy analysis would agree that it: (a) requires sensitivity to a given policy space, which is neither limited to a particular polity nor a decision-making system; (b) cannot be separated from politics; and (c) involves a practical motivation of careful evaluation of problems and, whenever possible, different solutions to these. The policy space for product safety is not limited to the EU’s parliamentary decision-making; national safety administrations and standardization organizations—among others—possess considerable power in both shaping and interpreting the overall safety policy. Such power leads to politics, which, in the present context, is also shaped by politicians as well as the organized interests of producers and consumers. Furthermore, in the EU, not only are legislations lobbied but interest group politics occur also in nonlegislative administrative institutions [39]. But in what follows, only a limited focus is placed on agency and the intentions of political actors. The identification of problems and bottlenecks satisfies the practical motivation, although policy recommendations are kept to a minimum as reforms are already underway in the EU.

Also the epistemological bases for policy analysis vary. At least interpretative, narrative, normative, critical, historical, positivist, evidence-based, and deliberative policy analysis frequently appear in the literature. The last one suits the purposes of this review well. Formulated in the early 2000s as a critical response to the distinctively positivist policy studies at the time, deliberative policy analysis builds on three pillars: interpretation, deliberation, and practice-orientation [30, 44]. Although a few descriptive statistics are presented, the analysis is based on a qualitative interpretation of the main policy artifacts, the safety legislations in the EU. In terms of deliberation and practice-orientation, the intention is to cover the main arguments in the historical and present policy debates, assessing the relative merits of these and giving a unique input through a synthesis.

Due to the deliberative approach, the focus is further on high-level issues and trends instead of legal, technical, and other nitty-gritties of some particular safety policies. In other words, the primary audience contains not only researchers and academia but also policy-makers and others on the European democratic fora. Because these political roundtables are in Europe, the review also excludes comparisons with other, non-EU, countries and international arrangements.

At the same time, the approach taken is a literature review. Quantitative reviews (such as meta-analysis) and protocol-based (such as systematic literature reviews) approaches do not go well with policy analysis. The reasons for this claim are many, ranging from a need to assess historical developments to the requirement to cover both politics and policies. Thus, a traditional, interpretative approach is followed with the literature; the goal is to develop a comprehensive understanding and critical assessment of existing knowledge via in-depth reading [5, 71]. This classical approach does not mean that the collection of literature would have been unsystematic. Many relevant databases were queried, among them ScienceDirect, HeinOnline, Taylor-Francis Online, SpringerLink, Wiley Online Library, IEEE Xplore, and SAGE Journals.

### Data

The few descriptive statistics presented are based on the EU’s Community Rapid Information System (RAPEX) [25]. Established in the early 2000s, RAPEX is a database for tracking notifications sent by national safety authorities about dangerous consumer products, excluding food and pharmaceutical products but including clothing, cosmetics, toys, electronic appliances, and many other product types. It is administrated by the European Commission like many analogous safety tracking systems, including those related medicines, drugs, food products, serious cross-border health threats, and chemicals incidents [17, 54]. Although a database was established already in the 1990s for alerts on dangerous consumer goods [66], it is no longer publicly available online; RAPEX provides records from 2005 onward. In total, $n=28,129$ entries were filed to it between 2015 and 19 January 2021.

**Fig. 1:**
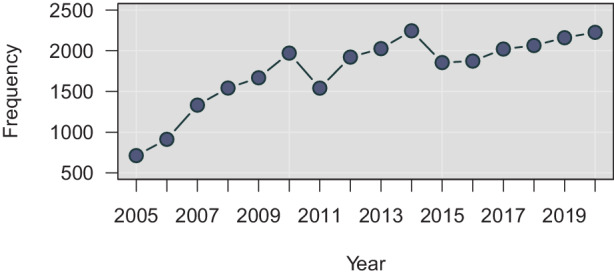
Annual Notifications (excluding 2021)

As can be concluded from Fig. 1, annual submission amounts have been relatively stable since the 2010s. After accelerating growth in the 2000s, roughly about two thousand entries were filed to RAPIX each year. It is difficult to interpret these magnitudes, but given the size of the EU’s internal market and the amount of consumer products circulating within and across it, a couple of thousand dangerous products per year seems modest.

## Review

The short review covers seven distinct but overlapping themes: the general, historical background, and the core regulations, administration, standardization, product categories, notifications, recalls, and liability, respectively.

### Background

Product safety is present in the Treaty on the Functioning of the European (TFEU). Like in many policy domains, the legal basis builds upon the functioning of the internal market for the free functioning of goods, people, services, and capital. In addition to this general clause specified in the TFEU’s Article 26, the member states have agreed upon the prohibition of import and export restrictions among themselves with Articles 34 and 35. However, the subsequent Article 36 states that indiscriminating restrictions are possible for goods on the grounds of protecting the health and life of humans, animals, or plants, among other things. Furthermore, Article 12 states a general goal of consumer protection when legislating and enforcing laws in the European Union, and Article 191 extends the overall health protection goal toward environmental considerations. Before the Lisbon Treaty in 2007, which incorporated the Maastricht Treaty as the TFEU, similar protective clauses were specified in the Treaty Establishing the European Community. As the European Economic Community was turning to the European Union, tighter harmonization was required also for product and food safety.

Instead of focusing on specific products, common European legislative framework was sought on four strategic areas: fair trading, public health, public controls, and consumer information, unified by standardization [11, 36]. These strategic goals were based on the so-called New Approach to regulation, which has generally been perceived as a success and thus an important factor for the European integration. In terms of jurisprudence, it started as a response to a seminal 1985 case in the Court of Justice of the European Union (CJEU). The decision reached by the court was significant for two reasons. Both were related to the internal market. On the one hand, it established the so-called principle of mutual recognition (i.e., goods sold in one member state must have access to the whole internal market); on the other, it mandated a set of essential public interest safety requirements for products [72]. The New Approach led to Directive 92/59/EEC on general product safety. It is based on five general principles, as follows:

1. The directive’s *scope* covers products intended for consumers, including new, used, or reconditioned products but excluding second-hand products. Safe products, in turn, refer to products that under normal, or reasonably foreseeable conditions of use, pose only a minimal risk. The antonym is a dangerous product. When assessing a risk, packing, instructions, and related factors should be taken into account in addition to a given product’s characteristics in itself.
2. The general safety *requirements* obligate producers to place only safe products on the internal market. While these are specified either by European standards or, in the absence of such standards, national laws enacted in the member states, compliance relies strongly on industry self-regulation and accreditation. In addition to these safety requirements, producers must provide adequate information to consumers and ensure that identification of individual products and product patches is possible after their release to the internal market.
3. The member states are obliged to ensure compliance through properly authorized national *authorities*. Their obligations range from compliance monitoring and safety checks to *ex ante* prohibitions for market entry and *ex post* withdrawal of products.
4. The member states are further mandated to provide *notifications* to the European Commission about any measures taken regarding dangerous products.
5. The *Commission* is empowered to inform other member states in case a given member state undertakes an emergency action for dangerous products. If a EU-wide solution is required, the Commission has also a right to enforce a withdrawal of a dangerous product. Finally, common EU institutions coordinate product safety issues between the member states.

The Maastricht treaty prompted an update to the product safety directive. In particular, the TFEU’s Article 169 strengthened the legal basis for consumer protection, including on health and safety issues. The resulting policy-making in the 1990s led to Directive 2001/95/EC, also known as the general product safety directive (GPSD). It is the directive in force today. Although the directive made many amendments and clarifications to the 1990s one, the five general principles remained largely unchanged. Among the amendments and clarifications are obligations for supply chain distributors of products. The GPSD also substantially extended the notification framework and information exchange procedures with the RAPEX architecture. Despite the architecture, further alterations were required for more efficient monitoring of the internal market. To this end, the 1990s Regulation (EEC) 339/93 was repealed with Regulation 768/2008 (hereafter, MSR) for more rigorous market surveillance of dangerous products. In general, the MSR strengthens the GPSD. A particular emphasis is placed on national accreditation authorities, serious risks, and further information exchange provisions.

Today, the GPSD and the MSR are the main effective legislations. That said, reforms were attempted throughout the 2010s, largely due to the emergence of electronic commerce. Already in 2013 a new regulatory package was attempted, but it got struck in a legislative limbo, as did the results from a 2016 evaluation by the Commission [72]. In practice, only the mutual recognition principle has been clarified with Regulation 764/2008 and its successor, Regulation (EU) 2019/515. Both had only a minimal impact upon the existing product safety legislations. A new consultation for product safety was launched in 2020 in a conjunction with larger planned reforms on electronic commerce, digitalization, and related aspects affecting the single market. Even though it is too early to evaluate the impact from the feedback, it is worth remarking that the political trenches were dug as could be expected. Regarding electronic commerce, platform companies, such as Ebay [19], argued that platforms should be exempted from tighter constraints, while consumer and civil society groups, such as BEUC [69], pointed out that a substantial amount of products purchased via platforms were already noncompliant with the EU laws and technical standards.

### Administration

The administrative framework is typical for the European Union in general; administration is decentralized to the member states. Within the safety domain, however, the decentralized framework is an exception because many other sectors (such as pharmaceuticals, transport, and aviation) are primarily administrated through specific EU agencies. In contrast, for product safety, the EU level is generally reserved for coordination and information exchanges. In fact, none of the articles in the TFEU establish a particular requirement for an EU-level competency. Instead, the legal basis for pan-European administration of product safety has largely been justified with the harmonization measures specified in the TFEU’s Article 114 [31, 59]. In practice, these measures include standardization and information exchanges. These were specified also in the GPSD and the MSR alongside the enforcement at the national level (Articles 6–10 in the former and Articles 2 and 16 in the latter). As the administration is typical to the EU, so are the impediments and problems.

To some degree, decentralized administration at the national level has maintained the historical cross-country differences and thus fragmentation across Europe. Depending on a study, three, four, or five different consumer protection regimes can be identified in Europe. According to one classification, there has been a Nordic *negotiation model* (industry associations and individual companies negotiate directly with consumer associations for common policy goals), a *protection model* with France as an example (consumer associations and the state have had a strong influence upon policy goals), and an *information model* with Austria and Germany as examples (industry associations and the state have had a dominant role) [1]. In addition, it is possible to identify further models by casting the focus on the British-influenced administrative tradition as well as the eastern and southern member states.

These regimes are not unique to product safety administration and consumer protection in general. For instance, a comparable administrative system—including the notification mechanism—has been established for European cyber security [64]. Also privacy and data protection in the EU share many similarities in terms of administration and coordination—as well as the associated problems [63]. These problems include a lack of resources, funding, and expertise in some countries, generally inconsistent enforcement, poor testing facilities in some countries, powerlessness in terms of sanctions, diverging legal interpretations, and general fragmentation [32, 42, 72, 73]. Given the Commission’s limited power—it cannot even act as an arbiter in case the member states disagree on product safety issues, many of the problems likely prevail in the foreseeable future. All this said, there has been a high degree of coherence for some consumer products due to standardization.

### Standardization

**Fig. 2:**
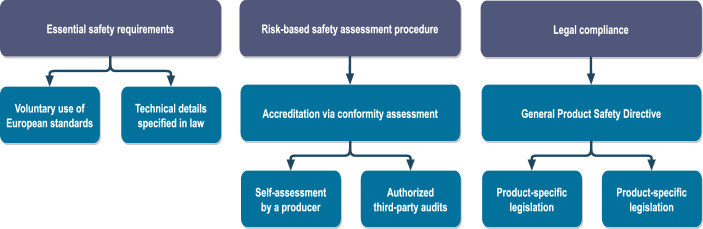
The General Logic of the Safety Policy in the European Union (adopted from [23] with alterations)

The EU legislations for product safety rely strongly on standardization. According to the New Approach, legislative harmonization establishes essential requirements required for an entry to the internal market, but discretion is allowed for producers regarding the technical standards that fulfill these requirements [15]. The role of standards in the safety policy’s general logic is illustrated in Fig. 2.

The difficulties of covering new products through regulation explain the difference between legislation and standardization. But the difference can be interpreted also as a way to separate politics from technical expertise, or, rather, to balance parliamentary legislation procedures with industry self-regulation [16, 38]. This balancing has also raised questions about the dominance of the latter over accountability [32]. In terms of legal wordplays, according to the GPSD, a product is *deemed* safe whenever it complies with a given European or national legislation. Yet, according to Articles 3 and 4 in the GPSD, a product is *presumed* safe insofar as it conforms to voluntary national standards transporting harmonized European standards. Here, European standards refer to those drafted by European standardization bodies in accordance with Directive (EU) 2015/153. It includes technical specifications that specify quality, performance, testing, safety, packaging, and related dimensions, but excludes radio and television broadcasting, telecommunications, and financial services, among other things. The major European standardization bodies for harmonized standards are the European Committee for Standardization (CEN), the European Telecommunication Standards Institute (ETSI), and the European Committee for Electrotechnical Standardization (CENELEC). All three are linked to international standardization organizations and their committees. Since the 2000s, European standardization efforts have generally leaned toward industry consortia and coregulation in order to improve competitiveness and innovation [4, 35]. Safety standardization is not an exception.

In some sectors, there has been some confusion between compliance to European standards and compliance to European safety legislations [45]. In many sectors, however, compliance questions are relatively straightforward for producers due to the work done by the European standardization organizations who translate the essential requirements into technical specifications. The CEN, in particular, has released thousands of technical standards over the decades. With these standards, accreditation provides the presumed safety conformity requirement [16]. The prime example is the CE marking established with Directive 93/68/EEC, which was later augmented with the Commission’s Decision 768/2008/EC. It is not a certification but a declaration of conformance and due diligence. Typically, the CE marking is obtained by self-certification and the following of harmonized European standards, with or without additional assessments by a national safety agency or third-party auditors [15, 57]. However, the procedure has been an exception rather than the rule for consumer products.

Fragmentation is present also in terms of standardization. Many complex products need to comply with multiple legislations *and* multiple standards. A further problem has been the GPSD’s generality; Article 3 implies that the presumed safety assumption rests on national standardization, which is required to transpose the various European standards. It is thus no wonder that the three European standardization organizations have called for unified EU-level standards, which, according to their position, are achievable via better coordination, funding, and strategic thinking [13, 20]. Finally, it is important to emphasize that compliance with European standards and their national transpositions is voluntary. While complying with these provides the presumed safety, it is still possible to place products that are only deemed safe onto the internal market. If the products turn out to be unsafe, withdrawal of these may follow by national authorities, as will soon be discussed.

### Product Categories

The New Approach pushed the regulatory work toward general product categories and essential safety requirements for these. A few notable legislations for product categories are enumerated in Table 1. These are the directives to which amendments were made with the CE-marking directive. Of these, the old voltage Directive 72/23/EEC is particularly noteworthy. It was this directive upon which the New Approach was largely modeled.

**Table 1:** Notable Safety Directives for Specific Products

Sector	Earliest legislation	Latest legislation
Burning gaseous fuels	Directive 90/396/EEC	Directive 2009/142/EC
Construction products	Directive 89/106/EEC	Regulation No 305/2011
Electrical equipment (voltages)	Directive 72/23/EEC	Directive 2014/35/EU
Electromagnetic compatibility	Directive 89/336/EEC	Directive 2004/108/EC
Hot-water boilers (fueling)	Directive 92/42/EEC	–
Implantable medical devices	Directive 90/385/EEC	–
Machinery	Directive 89/392/EEC	Directive 2006/42/EC
Nonautomatic weighing instruments	Directive 90/384/EEC	Directive 2014/31/EU
Personal protective equipment	Directive 89/686/EEC	Regulation (EU) 2016/425
Simple pressure vehicles	Directive 87/404/EEC	Directive 2014/29/EU
Telecommunications terminal equipment	Directive 91/263/EEC	Directive 93/68/EEC
Toys	Directive 88/378/EEC	Directive 2009/48/EC

Even with this small snapshot of the specific EU legislations, it can be concluded that the scope is wide, ranging from machinery and vehicles to medical devices and toys. Furthermore, many products (such as machinery or motor vehicles) must comply with multiple legislations [57]. It is also worth remarking that some notable consumer products, such as cosmetics [55], are missing from the listing. A further important point is that the relation of the specific legislations to the GPSD, and its predecessor has been a source of some confusion [35]. In principle, the GPSD and associated national laws should apply in the case when there are no specific legislations for a given product category. The MSR is also explicitly specified as *lex spesialis*; it is applicable only insofar as there are no specific legislations for market surveillance, as is the case with drug precursors, medical products, vehicles, and aviation. As the GPSD also states that the risks specified in it are applicable unless overruled by a specific legislation, it should be taken into account alongside any existing specific legislations.

**Fig. 3:**
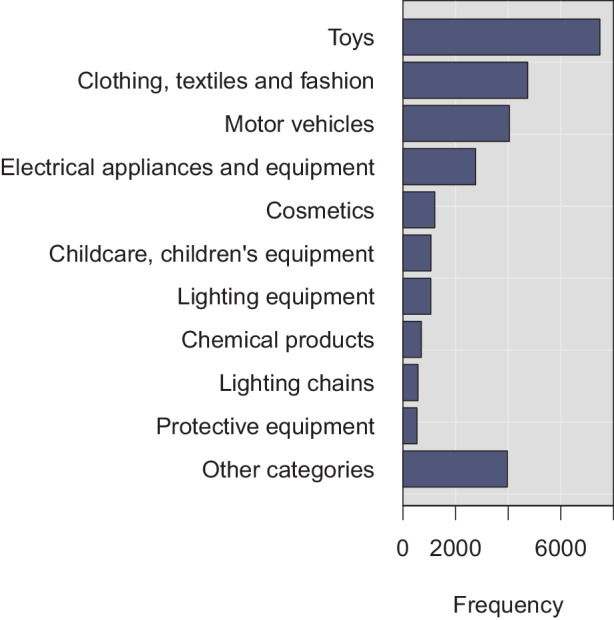
Top-10 Product Categories

To give us a sense of the actual, realized safety risks across the categories, Fig. 3 shows the ten most frequent product categories (alongside a catch-call group for other categories) across all entries filed to the RAPEX, from 2005 to mid-January 2021. Toys, clothing, and textiles, motor vehicles, and electronic devices constitute the majority of the reported unsafe consumer products. Together, these categories account for about 68% of all RAPEX filings. Particularly the large amount of dangerous toy products is interesting and surprising. It is, however, difficult to speculate about the reason for this result; one explanation could be that the safety of toys is vigorously enforced by the national safety authorities. This would align with the long tradition of considering children as a particularly vulnerable consumer group [52]. Numerous European standards have also been specified for toys [45]. In addition to toys, many filings were made about clothing, textiles, and fashion items, motor vehicles, and electrical appliances.

**Fig. 4:**
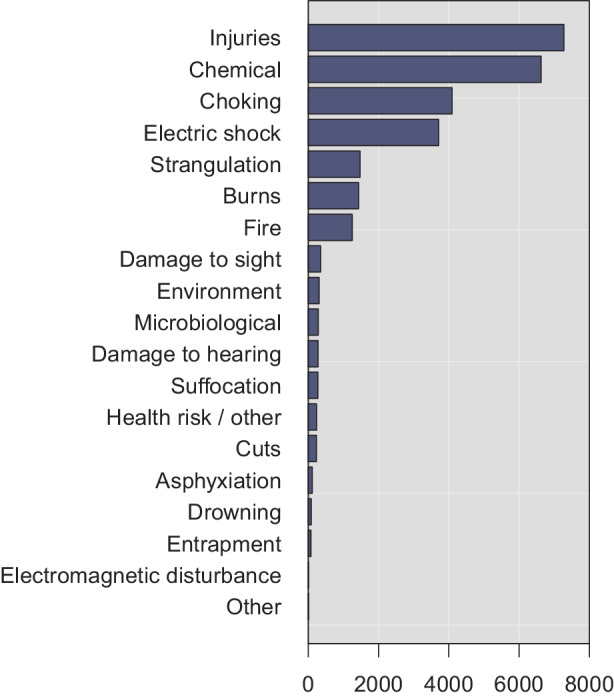
Risk Types

**Fig. 5:**
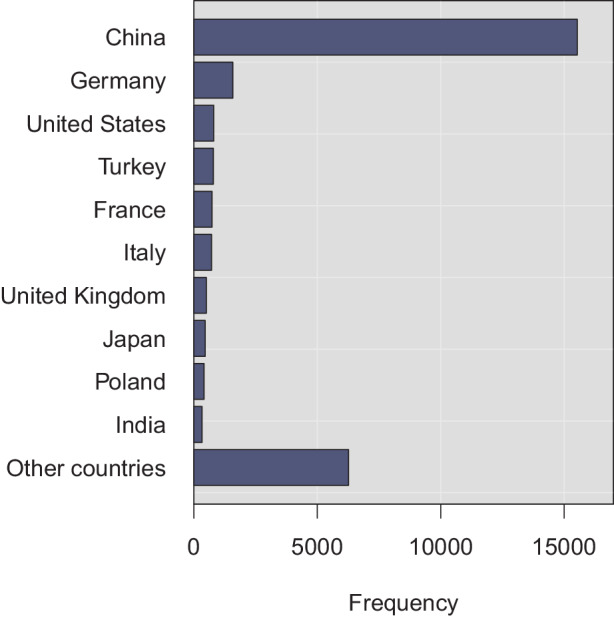
Top-10 Countries of Origin

Three additional points are worth making about the RAPEX entries. First: besides potential variance in terms of enforcement practices, these observations likely reflect the large amount of consumer products in falling to these categories. Second: the product categories further reflect the typical risk types shown in Fig. 4. For instance, about 34% and 32% of dangerous toy products caused chemical and chocking injuries, respectively. From all products with injury risks, about 41% were toys, 16% cosmetics, and 16% clothing, textiles, and fashion items. Last: the frequencies in Fig. 3 correlate with the origin from which the products were imported to the internal market. As can be seen from Fig. 5, the clear majority of dangerous products was imported from China. For instance, as much as 78% of the dangerous toy products were manufactured in China. As such, the observation cannot be strictly interpreted to imply that Chinese products would be particularly risky, given that most toy products are manufactured in China. Nevertheless—given that about 19% of total imports to Europe were from China in 2020 [26], it seems sensible to relate the observation to the increasingly complex supply chains for consumer and other products.

### Notifications

The notification mandates set in Directive 92/59/EEC were modeled after existing procedures laid down for some other sectors. Notably, notification procedures for pharmaceuticals were laid down already in the mid-1970s and early 1980s with Directives 75/319/EEC and 81/851/EEC. These were followed by Directives 82/894/EEC and 89/662/EEC concerning animal diseases and products of animal origin, respectively. Furthermore, a year after the Chernobyl disaster, Council’s Decision 87/600/Euratom established a system for rapid exchange of information in radiological emergencies. The GPSD generalized these procedures toward a general requirement: according to Article 5, both producers and distributors must inform a national authority whenever they know that a product contains safety risks and coordinate with them on any preventive measures taken. In practice, the mandate largely rests on producers’ own risk assessment and management procedures [35]. With accreditation, however, the national accreditation authorities should inform each other according to MSR’s Article 12. It is also worth mentioning notification requirements between the national authorities and the Commission, as well as information exchanges in situations requiring rapid interventions, as specified in the GPSD’s Article 11. Finally, there is the notification requirement toward the subjects being protected, the consumers; as specified in Article 16, information about safety risks should be available to the public according to transparency and other good administration practices. Some criticism has been leveraged about passivity in this informing obligation [31]. At the EU level, this criticism finds its target in the RAPEX infrastructure, as well as in its use by national authorities and media.

### Recalls

Product withdrawals were an important element already in the legacy Directive 92/59/EEC. Both the GPSD and the MSR clarified the powers granted to national authorities in this regard. The MSR’s Article 21 and the GPSD’s Article 5 stipulate that national authorities have the right to ban new market entries as well as withdraw existing dangerous products from the market. This withdrawal deterrence has provided an important incentive for producers to ensure safety of their products [12]. However, Article 5 in the GPSD somewhat loosens the obligations for producers by emphasizing that recalls should only be used as a last resort. This concession is understandable because recalls have been a controversial issue for both producers and regulators: for the former—besides plain economic losses, these may interfere with insurance schemes; for the latter, there has been a fear that producers will externalize difficult recall decisions to them [35]. In terms of product safety, however, withdrawals are particularly important, as these concern products that consumers are already using.

**Fig. 6:**
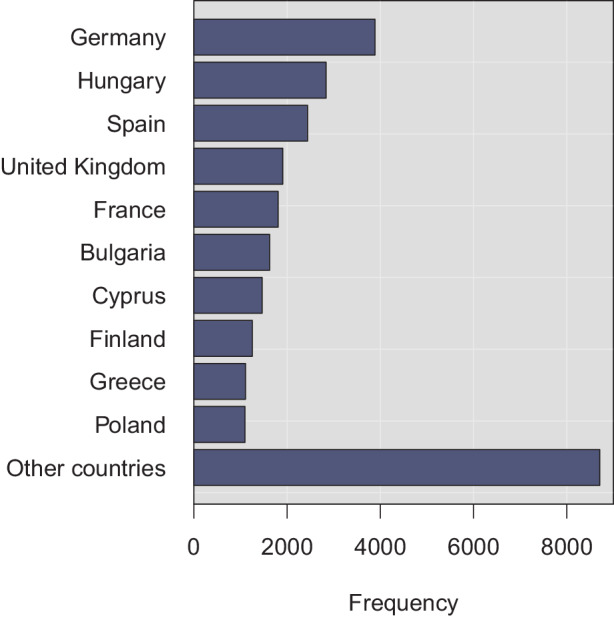
Top-10 Country Filings to RAPEX

Recalls have also been frequent according to the empirical RAPEX sample. By using simple regular expression searches, about 35% and 33% of the products’ descriptions contain the words *recall* and *withdraw*, respectively. Other keywords are far less common; these include *ban* (13%), *reject* (6%), *correct* (5%), and *destruct* (3%), among a few other auxiliary terms. Therefore, it can be concluded that the national safety authorities have also actively used their product recall powers. This observation reflects the many individual high-profile withdrawal cases with serious economic implications [61]. But RAPEX does not, unfortunately, contain enough information to deduce whether the many product withdrawals undertaken were done after a proactive notification from a producer or a distributor. Nor is it sufficient to deduce from the criticism that some countries have preferred voluntary corrective actions [73]. If filings to RAPEX is used as a proxy, a large cross-country variance is, indeed, present. For instance, more filings were made from Hungary than from France and the United Kingdom (see Fig. 6). This observation reinforces the earlier points about general fragmentation and incoherence. A further relevant question is whether the relatively low share of market entry bans indicates improvement potential for more proactive *ex ante* enforcement.

### Liability

A final important aspect of the New Approach is the adoption of the product liability Directive 85/374/EEC. It extends the hybrid approach of risk-based assessments, harmonization and standardization, and market surveillance with explicit allowance of litigation. Safety is explicitly spelled in the directive’s Article 6, and the legal basis for penalties is possible by translating the ‘‘polluter pays principle’’ implicitly present in the TFEU’s Article 191 on the safety context [59]. The directive introduced strict liability for producers, but there are several means by which a producer may argue against a claimant: improper use of the product in question, the presence of a defect at the time when the product was launched or marketed, the lack of scientific knowledge at the time when the defect was noticed, the presence of the defect despite compliance with legislations and standards, and so forth [60]. For these reasons, Directive 85/374/EEC cannot be fully interpreted to be a strict liability law—in practice, the necessity to demonstrate the damage, the defect, and the causal relation between them make the directive negligence liability law instead [27, 75]. It has nevertheless provided an incentive for producers to ensure safety [61]. All in all, it is important to stress that a ‘‘regulation through litigation’’ approach has generally been limited in the safety domain; reforms have been pushed forward by the organized interests of producers, consumers, and experts rather than by litigation, which is historically paradoxical due to the CJEU’s central role behind the New Approach [59]. Although hundreds of cases have been brought to courts throughout Europe [21], high-profile cases affecting large consumer groups have been rare for consumer goods.

There is no single explanation why the litigation-centric approach did not fully emerge. In addition to lobbying, the reasons include: the experiences in the United States, where strict liability was established for product safety in the 1960s but which came under criticism in the 1980s for its alleged economic impacts, the state-of-the-art defense provisions in Directive 85/374/EEC, the European tradition of compensations through welfare states, underdevelopment of insurance schemes for producers, a lack of contractual relationships in some cases, litigation costs for consumers, and the recall deterrence, among other things [7, 12, 21]. History offers another plausible explanation: Directive 85/374/EEC was enacted before there was any talk about the subsidiarity principle, before the Maastricht Treaty [27]. Regarding more recent times, an alternative interpretation would be that the GPSD, the auxiliary product-specific legislations, and European standards have worked efficiently in preventing dangerous goods from entering the internal markets to begin with. However, not all products are equally protected from safety threats. Although the question has been more about security than safety—a distinction, which, as noted, is not always clear, software liability has not generally progressed despite a long global debate [51, 65]. Europe is no exception [10]. As will soon be discussed, software and information technology products and services have also introduced other major security and safety challenges.

## Conclusion

This review addressed consumer product safety regulations in the European Union. Although the topic is complex—covering tens if not hundreds of legislations, many of which address highly specialized products and the science associated with them, some general points can be made briefly about the safety regulations. These are largely based on a hybrid approach. Thus, first, the GPSD and the MSR provide the umbrella legislations augmented with product-specific legislations and European standards. Both are relevant for compliance. Second, however, the hybrid policy is generally based on the precautionary principle. Although actual practice may be different, risk analysis carries a particular weight in the overall policy. Third, the administration is decentralized to the member states. Like in many related policy domains, the EU-level is mainly reserved for coordination and information exchanges. Fourth, even though possible, litigation has been relatively rare; product recalls remain the main deterrence.

Although recommending change for the sake of change is one of the deadly sins of policy analysis [49], there are many indicators of a reform need. Many of these have also been acknowledged in the EU. The Commission’s recent summary report [22] of the feedback on the GPSD suffice to outline the main factors behind the need. These are:

- Although the Commission pointed out *inconsistency* only in terms of food-imitating products, incoherence and fragmentation are a problem for the whole product safety policy. As is common in the EU, the reason is partially explained by both vertical (national policies versus EU policies) and horizontal (variance across the member states) incoherence. The issue goes beyond the safety legislations. For instance, the product liability directive has not been uniformly adopted in Europe [21]. Another notable aspect relates to European standards, which still need to be transposed to national standards. The position of European standardization organizations seems rather unequivocal: there is a need for unified standards at the EU level. Incoherence and fragmentation are not the only reasons; industry competitiveness is also a concern with standards. According to interviews [9], in some sectors, particularly small and medium-sided enterprises have faced challenges in selecting appropriate standards and implementing these for their products.
- *Enforcement* has been a closely related problem. Although the situation cannot be described as a race to the bottom whereby some member states would deliberately weaken their enforcement responsibilities [27], resources, expertise, legal interpretations, and incentives, among other factors, vary across the member states. As the Commission noted, the effectiveness of recalls is a particular concern because many consumers continue to use dangerous products despite recall notices and withdrawals of product stocks in sale. As for potential solutions, as the European Parliament emphasized in its 2019 resolution [24], further harmonization, sufficient resourcing, and better coordination may improve the situation. As there are EU agencies for safety in some sectors, it is also worth contemplating whether or not a move toward EU-level administration might offer a longer term solution.
- *Market surveillance* has been a third common problem. Closely related to the enforcement problems, according to the Commission, there has been a lack of tools and instruments to impose effective sanctions. As with security, the complex global supply chains constitute a major problem for product safety in Europe and elsewhere. Traceability, product integrity, and supply chain management are increasingly important for safety risk management and efficient recalls [46]. Analogous points apply to software products and their security [14]. Although consumer products may be an exception, it can be generally argued that European approaches are not sufficient alone; the management of large-scale hazards requires global solutions, and with such solutions, political obstacles should never be undermined [58]. Furthermore, within Europe, the enactment of the MSR has led to uneven requirements for different products; the incoherence across products and across Europe applies also to post-market activities. The same applies to RAPEX risk assessment guidelines, which are overly general and mainly left to national authorities [53]. The general fragmentation is again visible.
- *Online platforms* are an increasing problem for product safety and its enforcement. As such, the issue is hardly new. The difficulty to identify sellers and hold them accountable on electronic commerce marketplaces was recognized already in the early 2000s [7]. Yet, only recently have European safety authorities cast their attention to the issue. As an example: in total, since 2018, $244$ submissions to RAPEX have mentioned either eBay, Amazon, or both. Although the amount is tiny compared to the amount of consumer products delivered via these platforms, it is still sufficient to conclude that the problem is already recognized also on the side of enforcement. A particular problem with these new platforms relates to their business model; they act as intermediaries for third-party sellers whose responsibilities—or even identities—are not always clear. Like with supply chains, the issue is also largely global, and, unfortunately, existing international arrangements are soft guidelines at best in this regard [2]. As consumers increasingly purchase products from sellers located outside of the EU, ensuring safety has become more difficult for European authorities. Many of these products do not enter the internal market as large batches, which complicates the work at customs. In these cases, alerts are possible, but recalls are difficult.
- *New technologies* are the final problem recognized by the Commission—and for a good reason. Technologies such as artificial intelligence have fueled the whole safety debate to a new level throughout the world. But according to the Commission’s auxiliary report [23], the safety provisions for artificial intelligence applications, robotics, and Internet-of-things devices are seen as attainable mainly through the transparency, accountability, and unbiasedness of algorithms, fallback mechanisms, and keeping a human in the loop. Although these are sensible goals as such, it remains disputed how these could be legislated with sufficient rigor—especially when considering that safety science is not quite there yet [33]. For these and other reasons, liability for new technologies has been actively debated recently. Given that reforms are already underway on this front, it suffices to generally pinpoint the many challenges: unclear definitions, including the legal status of artificial intelligence systems [29], different legal notions of liability, potential (compulsory) insurance schemes, impacts upon economy and innovation, supply chains, and so on [8, 10, 75]. Another related point is the ethical use of artificial intelligence promoted by the EU. Here, it remains unclear whether ethics can—or should—be conflated with safety, which should arguably always be guaranteed regardless of what is seen as right or wrong.

Three additional points deserve a brief discussion. The first point is practical: the RAPEX system could benefit from more metadata. For instance, the textual descriptions often lack sufficient information to deduce which legislations or standards were specifically violated. Although some national authorities have occasionally used general phrasings such as ‘‘machine directive’’, most of the entries lack even such elementary cues. Some criticism has also been expressed previously regarding missing information about specific dangerous compounds in some products, and whether these ended at RAPEX due to producers’ self-disclosures or due to testing by national authorities [50]. Further small practical improvements are not difficult to imagine; there are no longer working hyperlinks pointing particularly to online platforms, and so on. In terms of bigger implementation challenges, integration with other safety alert systems remain a priority. Given the amount of consumer products imported from China (see Fig. 5), supply chain integration seems a particularly noteworthy goal. It may also provide synergies with security requirements for consumer products. These points call for comparative cross-country research addressing both technical aspects and policy challenges.

The second point is about the accreditation and its relation to other ongoing policy changes. Regulation (EU) 2019/881 established cyber security certification for information technology products. Even when keeping in mind that certification should not be equated with accreditation, the directive’s certification scheme is interesting because it differs from the New Approach tradition; it is not based on harmonized standards enacted by the three European standardization organizations, and, in some cases, certification via it may overlap with these standards and the product-specific legislations [43]. Besides aligning with security, the certification scheme largely builds on global soft guidelines augmented by the institutional European cyber security authority; a need for further harmonization and potential standardization is evident also in this regard [47]. From a policy perspective, further incoherence may thus follow in the long-run. This assertion leads us to consider the third and final point.

Last, it is sensible to contemplate whether the whole concept of safety should be defined better to reflect new realities. Many of the established demarcations seem outdated, both in academia and in practice. For instance, the distinction between products and services has acknowledgedly become blurry for legal, business, and safety considerations [3, 10, 21, 23]. Particularly information technology products have long relied on different services, nowadays often fueled by data. As the issues with global supply chains demonstrate, it is also increasingly difficult to make clear-cut distinctions between producers, suppliers, and distributors. When data powers capitalism, even consumers are producers, suppliers, and distributors themselves; they produce, supply, and distribute their data for the new means of production [62, 70]. Then, there is the safety concept itself. The relation between security and safety has always been ambiguous. Privacy as a safety concern has also long been debated [28]. But there is more. Even information itself can today be seen as a safety issue—if not a hazard. For instance, recent results indicate a substantial amount of vaccine misinformation (some of which may also be intentional disinformation) associated with potentially dangerous products sold on electronic commerce platforms [41]. These and other similar results restate the general platform problem. But they further call us to theorize and reconceptualize safety.
